# A deceptive Nonhealing scalp ulcer revealing cutaneous angiosarcoma

**DOI:** 10.1016/j.abd.2026.501477

**Published:** 2026-10-28

**Authors:** Ilse Yolanda Osorio Aragón, Jorge Alberto Cortez Vila, María Elisa Vega Memije, Roberto Arenas Guzmán, Sonia Toussaint Caire

**Affiliations:** a Department of Dermatopathology, Dr. Manuel Gea González General Hospital, Mexico City, Mexico; b Department of Dermatology, Dr. Manuel Gea González General Hospital, Mexico City, Mexico; c Department of Mycology, Dr. Manuel Gea González General Hospital, Mexico City, Mexico

Dear Editor,

Cutaneous Angiosarcoma (CAS) is an aggressive soft tissue sarcoma that represents 1%-2% of them; it is composed of endothelial cells of blood or lymphatic vessels.^1^

It typically presents as multiple raised purplish plaques, papules, or nodules, with a variable growth rate. High-grade lesions often tend to ulcerate. When located on the scalp, alopecia is usually absent.^2^ In this case, we describe an unusual presentation as a solitary ulcerated lesion on the scalp.

A 59-year-old man presented with a 3-month history of a 6 × 3 cm oval ulcer with a friable surface and borders, associated with meliceric crusts and a positive pull test (Fig. 1).

**Figure 1:**
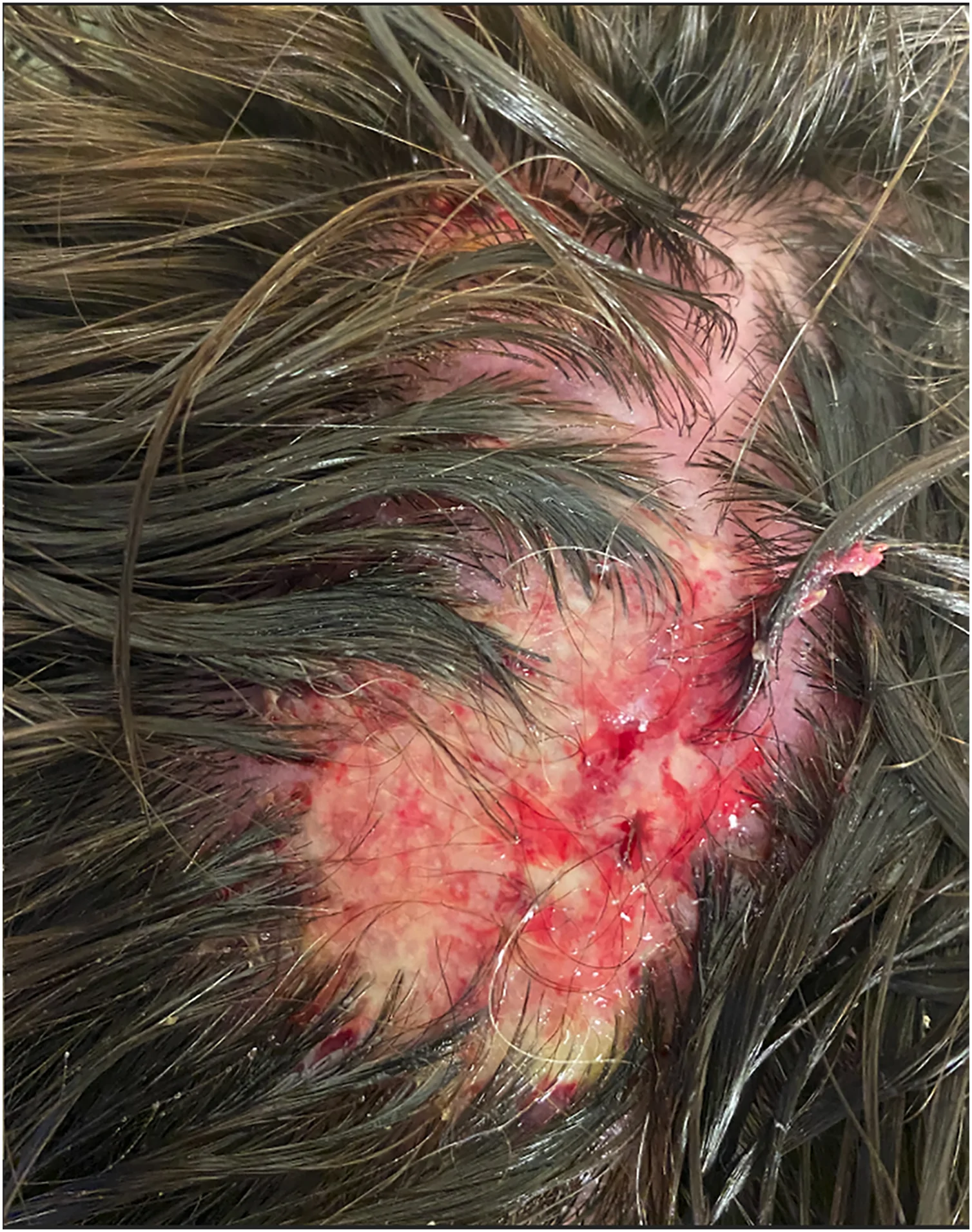
Clinical photograph showing an oval-shaped ulcer measuring approximately 6 × 3 cm.

The patient had received multiple treatments without improvement. He was subsequently referred to our institution for further evaluation. Previous histopathological slides were reviewed, and a new skin biopsy was performed.

The histopathological analysis revealed an epidermis exhibiting focal ulceration. The dermis showed a biphasic tumor pattern. Papillary and reticular dermis (Fig. 2) featured a proliferation of atypical, pleomorphic epithelioid cells with centrally located nuclei, dense chromatin, and conspicuous nucleoli. Interspersed erythrocytes were noted within this cellular infiltrate. The reticular dermis and subcutis (Fig. 3) demonstrated an irregular, infiltrative pattern characterized by multiple cleft-like vascular spaces that were lined by atypical endothelial cells that projected into the lumen and contained erythrocytes. The cells displayed pleomorphism, atypical mitoses, and prominent nuclei and nucleoli. The malignant vascular channels were observed to extend into the subcutaneous tissue, surrounding pre-existing blood vessels (promontory sign) and adnexal structures. Immunohistochemistry revealed positivity for CD31, D2-40, and ERG (Fig. 4), confirming the diagnosis of CAS.

**Figure 2:**
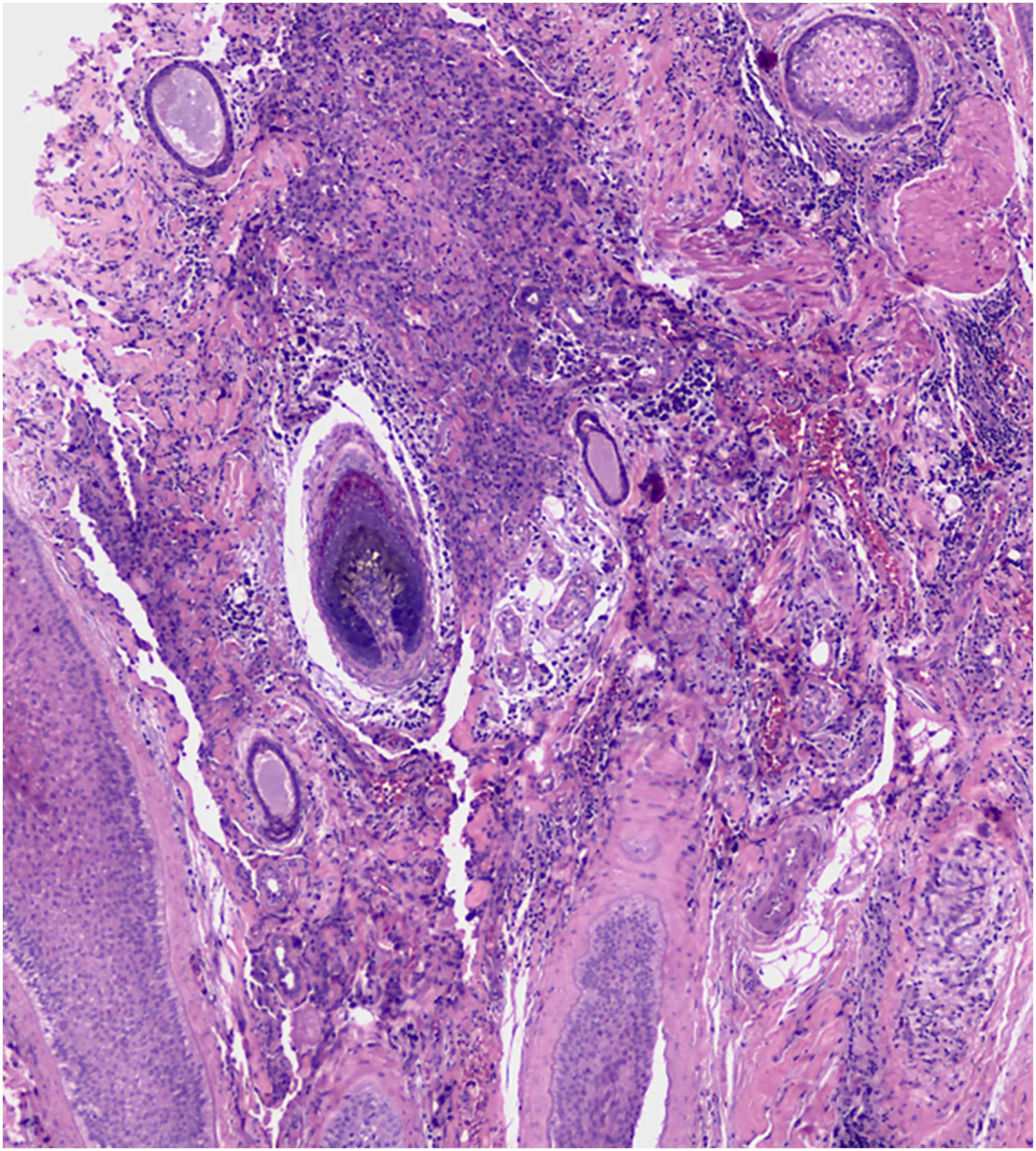
Superficial dermis showing a proliferation of atypical pleomorphic epithelioid cells with centrally located nuclei, dense chromatin, and prominent nucleoli. Interspersed erythrocytes are present within the infiltrate.

**Figure 3:**
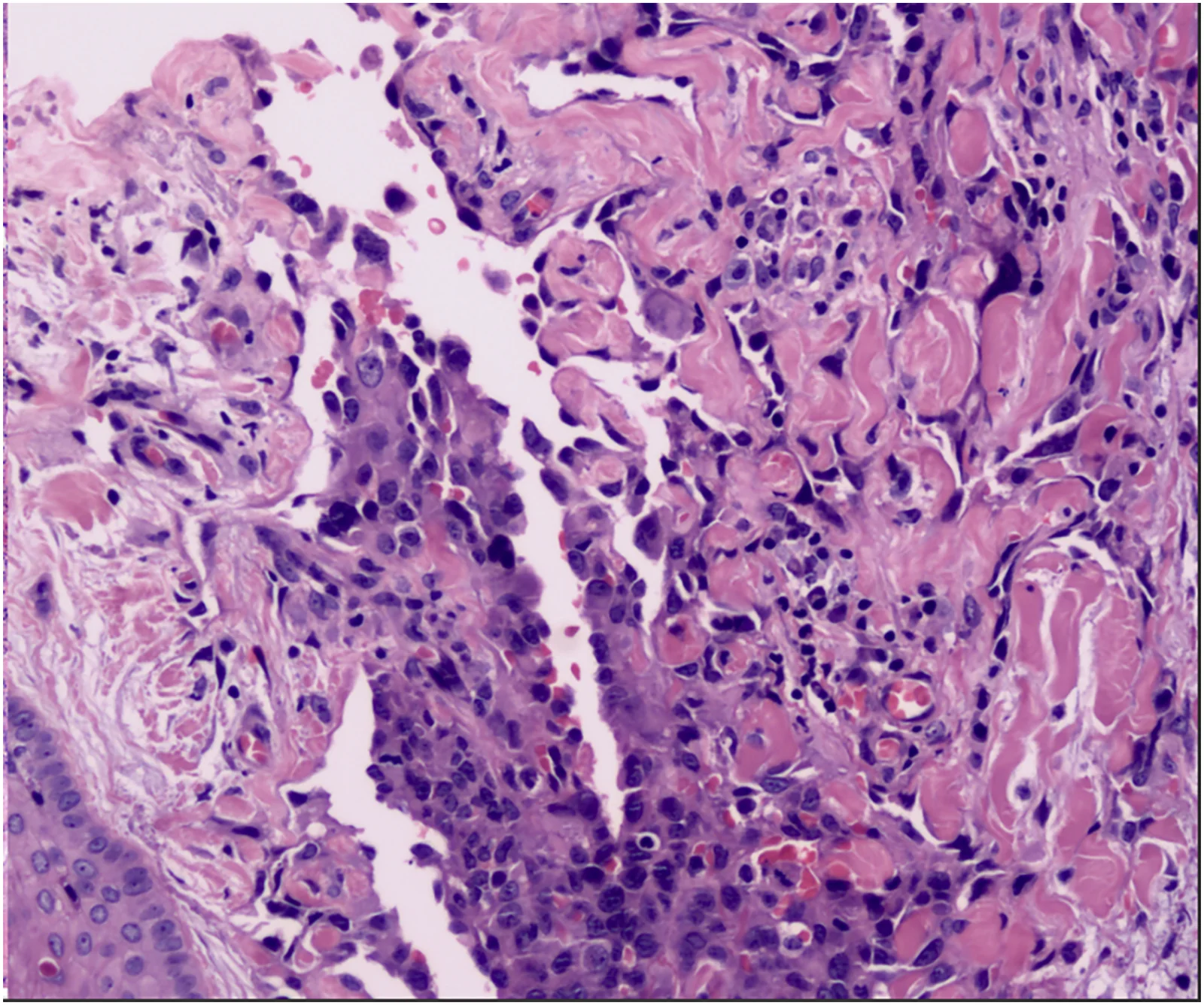
Mid to deep dermis and subcutis demonstrating an irregular infiltrative pattern with multiple cleft-like vascular spaces lined by atypical endothelial cells projecting into the lumen and containing erythrocytes.

**Figure 4:**
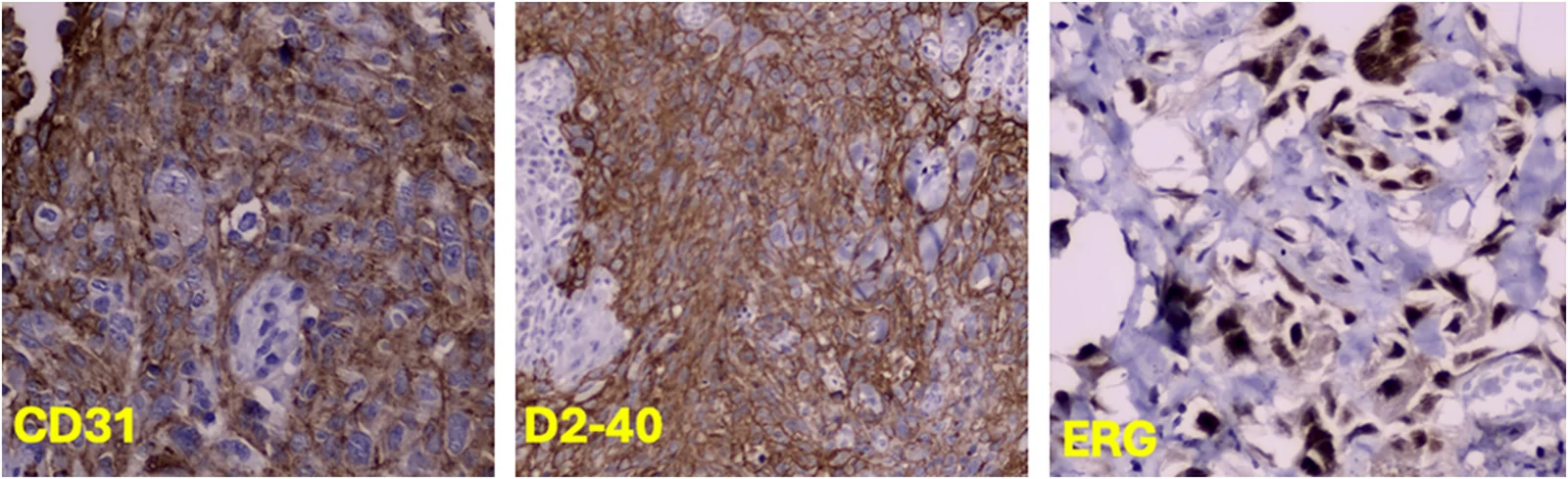
Immunohistochemical staining showing diffuse membranous positivity for CD31 and D2-40, with nuclear positivity for ERG.

CAS is an uncommon skin malignancy that usually affects Caucasian men over 60-years old. It can present in two distinct forms. The primary form arises without a clearly identifiable cause, although associations have been reported with conditions such as xeroderma pigmentosum, epidermolysis bullosa, Klippel-Trenaunay syndrome, HIV, and post-renal transplantation. The secondary form is most often related to prior breast radiation or chronic lymphedema of the breast and upper limb.^2^, ^3^

It commonly presents as a purple-reddish plaque, papule or nodule in the face or scalp; as the tumor gets bigger, oedema, tissue infiltration, and ulceration may be seen. Unfortunately, the lesion tends to be bigger than it appears.^3^ However, CAS may present with atypical features that make diagnosis challenging. Based on the epidemiological profile and the clinical presentation, our differential diagnosis suggested, as an alternative, a papulopustular erosive scalp dermatitis, Brunsting-Perry pemphigoid, or even an aggressive squamous cell carcinoma.

According to Kostaki et al.,^4^ CAS can mimic a broad spectrum of conditions, including inflammatory diseases (eczema, rosacea, sarcoidosis, Morbihan disease), autoimmune disorders (Lupus, connective tissue diseases, hypothyroidism), infections (herpes zoster, cellulitis, chronic ulcers), and other tumors (Kaposi sarcoma, hemangiomas, hematomas). However, the authors also noted that epidemiological characteristics ‒ such as age, sex, and tumor location ‒ do not appear to differ between patients with typical presentations and those with atypical forms. Time to diagnosis ranges from 1 month to 7-years, with most cases diagnosed after 3-months, similar to our case. Scalp involvement is associated with longer delays, likely due to difficulty in recognizing and visualizing lesions.^5^

Histological examination is essential to confirm the diagnosis. CAS is characterized by anastomosing vascular channels lined by prominent, pleomorphic endothelial cells with evident mitotic activity. It may exhibit papillary projections or cellular nests within the vascular lumen, and vascular proliferation often dissects the surrounding collagen fibers. The tumor can display epithelioid features, as well as solid, undifferentiated, or spindle cell morphology. Reticulin staining helps confirm vascular origin. Immunohistochemistry typically shows positivity for CD31, ERG (most sensitive and specific), and D2-40, with CD34 often negative.^2^

Management remains challenging and non-standardized. In non-metastatic disease, surgical excision is associated with improved survival, whereas it offers no benefit in metastatic cases. Adjuvant options include radiotherapy, chemotherapy (e.g., paclitaxel), and targeted or immunotherapies such as bevacizumab and pembrolizumab, with variable outcomes.^6^

Our case highlights how atypical presentations can lead to misdiagnosis and delayed treatment. It underscores the need to maintain a high index of suspicion for CAS, particularly given its ability to mimic a wide range of conditions and the diagnostic delays frequently reported.

## ORCID ID

Ilse Yolanda Osorio Aragón: 0000-0002-7134-026X

María Elisa Vega Memije: 0000-0001-7985-118X

Roberto Arenas Guzmán: 0000-0002-2992-9564

Sonia Toussaint Caire: 0000-0002-6883-3100

## Research data availability

Does not apply.

## Financial support

This study was not supported by any sponsor or funder.

## Authors’ contributions

Ilse Yolanda Osorio Aragón: Data collection, analysis and interpretation; manuscript critical review; preparation and writing of the manuscript; study conception and planning.

Jorge Alberto Cortez Vila: Data collection, analysis and interpretation; manuscript critical review; preparation and writing of the manuscript; study conception and planning.

María Elisa Vega Memije: Effective participation in research orientation; study conception and planning.

Roberto Arenas Guzmán: Effective participation in research orientation; manuscript critical review; preparation and writing of the manuscript; study conception and planning.

Sonia Toussaint Caire: Effective participation in research orientation; study conception and planning.

## Conflicts of interest

None declared.
